# Metabolite Profiling and Identification of Sweet/Bitter Taste Compounds in the Growth of *Cyclocarya Paliurus* Leaves Using Multiplatform Metabolomics

**DOI:** 10.3390/foods13193089

**Published:** 2024-09-27

**Authors:** Liang Chen, Dai Lu, Yuxi Wan, Yaqian Zou, Ruiyi Zhang, Tao Zhou, Bin Long, Kangming Zhu, Wei Wang, Xing Tian

**Affiliations:** 1 TCM and Ethnomedicine Innovation & Development International Laboratory, Innovative Material Medical Research Institute, School of Pharmacy, Hunan University of Chinese Medicine, Changsha 410208, China; chenliang_201230@163.com (L.C.); x944084134ld@163.com (D.L.); wanyuxi2005@163.com (Y.W.); zouyaqian2003@163.com (Y.Z.); zhangry2003812@163.com (R.Z.); zhoutao20021108@163.com (T.Z.); 19873178048@163.com (B.L.); wangwei402@hotmail.com (W.W.); 2 Department of Food and Drug Engineering, School of Pharmacy, Hunan University of Chinese Medicine, Changsha 410208, China; 3 School of Informatics, Hunan University of Chinese Medicine, Changsha 410208, China; 13526009623@163.com; 4 Hunan Engineering and Technology Research Center for Health Products and Life Science, Changsha 410208, China

**Keywords:** *Cyclocarya paliurus* leaves, sweet/bitter/bittersweet, nontargeted metabolomics, electronic tongue, molecular docking

## Abstract

*Cyclocarya paliurus* tea, also known as “sweet tea”, an herbal tea with *Cyclocarya paliurus* leaves as raw material, is famous for its unique nutritional benefits and flavor. However, due to the unique “bittersweet” of *Cyclocarya paliurus* tea, it is still unable to fully satisfy consumers’ high-quality taste experience and satisfaction. Therefore, this study aimed to explore metabolites in *Cyclocarya paliurus* leaves during their growth period, particularly composition and variation of sweet and bitter taste compounds, by combining multi-platform metabolomics analysis with an electronic tongue system and molecular docking simulation technology. The results indicated that there were significant differences in the contents of total phenols, flavonoids, polysaccharides, and saponins in *C. paliurus* leaves in different growing months. A total of 575 secondary metabolites were identified as potential active metabolites related to sweet/bitter taste using nontargeted metabolomics based on UHPLC-MS/MS analysis. Moreover, molecular docking technology was utilized to study interactions between the candidate metabolites and the sweet receptors T1R2/T1R3 and the bitter receptors T2R4/T2R14. Six key compounds with high sweetness and low bitterness were successfully identified by using computational simulation analysis, including cis-anethole, gluconic acid, beta-D-Sedoheptulose, asparagine, proline, and citrulline, which may serve as candidates for taste modification in *Cyclocarya paliurus* leaves. These findings provide a new perspective for understanding the sweet and bitter taste characteristics that contribute to the distinctive sensory quality of *Cyclocarya paliurus* leaves.

## 1. Introduction

*Cyclocarya paliurus (Batalin) Iljinskaja* (*C. paliurus*), also known as “sweet tea tree” and “the third tree”, is a deciduous tree belonging to the genus *Cyclocarya Iljinskaja (Juglangdaceae)* and is widely distributed in south China, with the southwestern area of Hunan province being one of its important native habitats [1]. As a traditional edible and medicinal plant, *C. paliurus* leaves taste sweet, often as a “sweet tea” traditionally consumed daily by the local Chinese populace for more than 1000 years, and its safety and flavor have long been recognized [2]. As a traditional Chinese medicine, the leaves of *C. paliurus* contain rich biological active substances, such as flavonoids, terpenoids, sterols, acids, glycosides, and trace elements [3], for the treatment of diabetes, hypertension, and hyperlipidemia, as well as for reducing cholesterol and modulating functions of the immune system [4]. However, limited information is available about acute toxicity of extracts from *C. paliurus* leaves [5]. In 2013, *C. paliurus* leaves were approved by the National Health and Family Planning Commission as a new food ingredient, which can be eaten or drunk as ordinary food [6]. Recently, tea processed from *C. paliurus* leaves has become the first health tea approved by the US Food and Drug Administration in China and has been industrialized, which has broad market prospects [7]. Of note, despite the many reputations and valuable effects of *C. paliurus* leaves, due to the widespread coexistence of “sweetness and bitterness” (sweetness and bitterness are often perceived at the same time during drinking *C. paliurus* tea ), they are still unable to deliver an exceptional taste experience that meets consumers’ expectations for quality and satisfaction, resulting in limited product market recognition [8]. In fact, research on processing and utilization of *C. paliurus* leaves mainly focuses on volatile compounds and biological activities, while research on sweet and bitter substances in *C. paliurus* leaves has not been reported [9]. Therefore, exploring the potential “sweet and bitter” substances in the leaves of *C. paliurus*, and further screening the key taste substances, will definitely contribute to expansion of the application field of the whole industrial chain of *C. paliurus*, which has important social value and economic benefits.

“Bitterness” and “sweetness” are often functionally and metaphorically opposed, and this antagonism is complicated by the fact that some compounds exhibit both sweet and bitter tastes. In fact, in previous reports the sweet/bitter substances in plants were mainly composed of terpenoids (diterpenoids or triterpenes), flavonoids, glycosides, sweet/bitter amino acids, and phenolic acid [10,11]. Detection of sweet/bitter compounds in foods or medicinal and edible plants has traditionally relied on sensory-guided fractionation via preparative chromatography; however, use of this method has been limited because it is time consuming and laborious [12]. Thus, there is an urgent need to use faster and more efficient methods to screen sweet/bitter/bittersweet substances in plants. Moreover, few studies [3,13] have been conducted on the core flavor metabolites that influence the sensory qualities of *C. paliurus* leaves.

As a rapid and repeatable high-throughput detection technology, metabolomics analysis can identify a wide variety of metabolites in biological samples and has been widely used to investigate formation and transformation of various intracellular metabolisms and compounds in plants [14]. The electronic tongue system, as an intelligent instrument that simulates human taste, has been reported to be able to quantitatively and qualitatively analyze the taste of different medicinal and edible plants [15,16]. Generally, nontargeted metabolomics has been used to assess the relationship between metabolites and sensory quality, such as in identifying key taste compounds in food, combined with sensory evaluation and intelligent taste analysis [12]. Moreover, databases related to food flavor have also been established in recent years, such as VirtualTaste and BitterDB [17,18]. Thus, LC-MS-based nontargeted metabolomics combined with electronic tongue analysis is an excellent method to establish the relationship between *C. paliurus* leaves’ core metabolites and sweetness/bitterness/bittersweetness. 

A number of studies have focused on binding interactions between key taste compounds and taste receptors [19]. Sweet/bitter receptors play an irreplaceable role in the process of taste perception, and their structure and function has become a new research focus [20]. The perception of bitterness and sweetness stems from complex interactions between compounds and receptors. Sweetness begins with interaction between the sweet-active substance and a pair of special receptors (T1R2/T1R3) located on taste buds. Therefore, the perception of sweetness is extremely low when T1R2 or T1R3 is present alone, but once the two monomers are combined, taste receptor cells can directly convert information content of chemical stimulation into neural signals and transmit them to the brain, which can perceive almost all sweet tastes [21,22]. Bitter taste receptors (hT2R4 and hT2R14) are known to be activated by a large number of compounds originating from different chemical categories [23,24]. In fact, in silico molecular modeling techniques have been successfully used to screen and evaluate the binding mechanisms of interesting compounds to the taste receptors, including sweet and bitter receptors [19,22].

The chemical composition, metabolites, and taste of *C. paliurus* leaves may vary somewhat depending on its growth period. Thus, the present study aimed to investigate the flavor-related metabolites that contributed to the sweet/bitter/bittersweet taste of *C. paliurus* leaves harvested in different growing months by using multi-platform metabolomics analysis, including the electronic tongue system, ultra-performance liquid chromatography/tandem mass spectrometry (UHPLC-MS/MS), and molecular docking for metabolite profiling. This approach is expected to provide new insights into the key taste components of *C. paliurus* leaves and contribute to development of strategies for enhancing the sensory quality of their products.

## 2. Materials and Methods

### 2.1. Reagents and Chemicals

The samples of *C. paliurus* leaves from various growth months were procured from Hunan Yueling Junshan Agroforestry Technology Co., Ltd. (Hunan, China). Folin–Ciocalteu’s phenol reagent was obtained from Hefei Bomei Biotechnology Co., Ltd. (Hefei, China). Anhydrous ethanol was acquired from Anhui Ante Food Co., Ltd. (Anhui, China). Phenol was purchased from Guangdong Guanghua Technology Co., Ltd. (Guangdong, China). Sulfuric acid and perchloric acid were obtained from Hunan Huihong Reagent Co., Ltd. (Hunan, China). Gallic acid, rutin solution, glucose, and oleanolic acid were guaranteed reagents provided by Chengdu Lemeitian Pharmaceutical Technology Co., Ltd. (Chengdu, China). Anhydrous sodium carbonate, sodium nitrate (III), aluminum nitrate(V), vanillin, and glacial acetic acid were sourced from Sinopharm Chemical Reagent Co., Ltd. (Shanghai, China).

### 2.2. Cyclocarya Paliurus Samples

All samples of *C. paliurus* leaves were collected from the Huping Mountain in Shimen County, Hunan, P.R. China (110.773665° N, 29.94036696° E). To investigate the cumulative dynamics of substances in the leaves of *C. paliurus* throughout its growing period, approximately 200 ± 0.50 g of fresh, fully mature leaves was collected at four-week intervals, starting from May and ending in September 2023 (1 May, 1 June, 1 July, 1 August, and 1 September). These samples were randomly gathered from three trees with similar canopy characteristics and were mixed to create a pool representing each month. All samples were dried in an oven at 60 °C until a constant weight was achieved. Subsequently, the *C. paliurus* leaf samples were ground into powder and refrigerated at −20 °C for further analyses.

### 2.3. Electronic Tongue Analysis for C. Paliurus Samples

Taste attributes (saltiness, bitterness, sourness, umami, umami aftertaste, astringency, bitter aftertaste, and astringency aftertaste) of the five different growth months of *C. paliurus* leaf samples were determined using a TS-sa402b electronic tongue system (Intelligent Sensor Technology Co., Ltd., Atsugi, Japan) with wide area selection specific artificial lipid membrane sensors. The taste attribute indices for the tested samples were calculated based on the absolute value of the lipid membrane potential for each artificial sensor, referenced against the potential of the solution. The output value of the reference solution was considered the tasteless point, set at 0 for different taste components, and values bigger than these tasteless points were considered meaningful. A precisely weighed (2.00 ± 0.05) g *C. paliurus* leaf sample was steeped in 150 mL of boiling water for 1 h. Subsequently, the aqueous extract was filtered through three layers of gauze and allowed to cool to 25 °C before undergoing analysis using the TS-sa402b electronic tongue system. Each sample was cycled 4 times, and the mean values of the last three cycles were used for statistical analysis using TS-sa402b Library search software (Intelligent Sensor Technology Co., Ltd., Atsugi, Japan). 

### 2.4. Determination of Total Phenolic Content (TPC), Total Flavonoid Content (TFC), Total Polysaccharide Content (TP), and Total Saponin Content (TSC)

To determine total polyphenols, total flavonoids, total polysaccharides, and total saponins, 6.0 ± 0.01 g of each *C. paliurus* leaf-powder sample was mixed with 120 mL of distilled water in a 250 mL round-bottomed flask and extracted at 90 °C for 40 min in a water bath. After extraction, each extracted solution was centrifuged at 4,500 rpm for 5 min at 4 °C. Then, all the resulting water extract was filtered through three layers of gauze three times to obtain the filtrate. The filtrate was concentrated under reduced pressure (75 kPa) and a temperature of 65 °C, followed by freeze-drying using a freeze dryer (Xinzhi freeze-drying equipment Co., LTD, Ningbo, China). After drying, the lyophilized *C. paliurus* powder was obtained, sealed, and refrigerated at −20 °C for further analyses. The TPC, TFC, TP, and TSC were determined using lyophilized *C. paliurus* powder. The extraction yield was calculated as the percentage of the initial fresh weight to the final dry weight after lyophilization, and all samples were analyzed in parallel three times.

The TPC in the *C. paliurus* leaf samples was determined by using the Folin–Ciocalteu colorimetric method, as described by Musci et al [25]. Specifically, an aliquot of 250 μL of sample infusion (solid–liquid ratio: 1:100) or gallic acid standard solution were pipetted into a cuvette containing 1 mL of Folin–Ciocalteu reagent. After 5 min at room temperature, 1 mL of 10% Na_2_CO_3_ was added. The absorbance was measured at 765 nm against a blank sample. The TPC was calculated based on the standard curve using gallic acid and represented as gallic acid equivalents (GAE) mg/g of leaves on dry weight. 

The TFC was assayed using the aluminum chloride colorimetric technique in accordance with Yang et al. [26]. Specifically, 2 mL of tea extract was mixed with 6.4 mL of a 1.5% AlCl_3_ solution and 3.2 mL of sodium acetate buffer solution (0.1 M, pH 5.5). The combined volume was brought up to 20 mL with 50% ethanol, incubated in the dark at 25 °C for 30 min, and the absorbance was measured at 415 nm. A standard curve was prepared using different concentrations of rutin solution, and the results were expressed as rutin equivalents (RE) mg/g of leaves on dry weight. 

The TP was analyzed using the phenol–sulfuric acid method [27]. A total of 1.0 g lyophilized powder was mixed with 20 mL of distilled water and extracted twice at 90 °C for 2 h. After extraction, the filtrate was adjusted to constant volume 50 mL. After diluting 50 times, 1 mL of diluted test samples was taken out, and 1 mL of 5% phenol and 5 mL of concentrated sulfuric acid were mixed. The mixture was incubated in a water bath at 40 °C for 30 min. After cooling, the absorbance of the sample was measured at 490 nm. The results were expressed as glucose equivalents (GE) mg/g of leaves on dry weight. 

The detection method for the TSC was as referred to by de Aguiar NS [28]. Using the spectrophotometric method, it is possible to quantify TSC relative to the triterpene nucleus released after a hydrolysis reaction, with subsequent reaction with vanillin. Additionally, a standard curve was prepared using different concentrations of oleanolic acid, and the results were expressed as oleanolic acid equivalents (OAE) mg/g of leaves on dry weight.

### 2.5. Nontargeted Metabolomics Analysis by UHPLC-MS/MS

#### 2.5.1. Metabolite Extraction

Fifty milligrams of each *C. paliurus* leaf sample was accurately weighed. Metabolites were extracted using 1 mL precooled mixtures of methanol, acetonitrile, and water (v/v/v, 2:2:1) and then placed for 1 h of ultrasonic shaking in ice baths. Following sonication, the mixture was placed at −20 °C for 1 h and centrifuged at 14,000 rpm for 20 min at 4 °C. The supernatants were carefully recovered and concentrated to dryness under vacuum conditions. To prepare the samples for mass spectrometry, 150 µL of precooled methanol–water (4:1, v/v) solution was added to redissolve the dried sample. The resulting mixture was centrifuged at 20,000 rpm for 20 min at 4 °C, and the supernatant was collected for further analysis.

#### 2.5.2. UHPLC-MS/MS Analysis

The metabolomics profiling was conducted using a UPLC-ESI-Q-Orbitrap-MS system (UHPLC, Shimadzu Nexera X2 LC-30AD, Shimadzu, Japan) coupled with a Q-Exactive Plus instrument (Thermo Scientific, San Jose, CA, United States of America).

For liquid chromatography (LC) separations, samples were analyzed using an ACQUITY UPLC® HSS T3 column (2.1 × 100 mm, 1.8 μm). The flow rate was 0.3 mL/min, and the mobile phase consisted of: A: 0.1% formic acid (FA) in water and B: 100% acetonitrile (ACN). The gradient was 0% buffer B for 2 min and was linearly increased to 48% in 4 min and then up to 100% in 4 min and maintained for 2 min before decreasing to 0% mobile phase B in 0.1 min.

For mass spectrometry (MS) data acquisition, electrospray ionization (ESI) was performed with positive and negative modes applied separately. The following HESI source conditions were set: spray voltage: 3.8kV (positive) and 3.2kV (negative); capillary temperature: 320 °C; sheath gas (nitrogen) flow: 30 arb (arbitrary units); aux gas flow: 5 arb; probe heater temp: 350 °C; S-Lens RF Level: 50. The instrument was set to acquire data over the *m/z* range of 70-1050 Da for full MS scans. Full MS scans were acquired at a resolution of 70,000 at *m/z* 200, with a maximum acquisition time of 100 ms for MS scans and 50 ms for MS/MS scans. The isolation window for MS2 was set to 2 *m/z*, and the normalized collision energy (stepped) was set as 20, 30, and 40 for fragmentation.

#### 2.5.3. Metabolome Data Preprocessing

The raw mass spectrometry (MS) data were processed using data-independent MS/MS deconvolution for comprehensive metabolome analysis; (MS-DIAL), a software tool for data-independent analysis (DIA) in mass spectrometry, was utilized for the analysis of the samples [29], for peak alignment, retention time correction, and peak area extraction. The identification of metabolites was based on accurate mass (tolerance < 10 ppm) and MS/MS data (tolerance < 0.02 Da) matched against databases such as HMDB (https://hmdb.ca/ (accessed on 9 March 2024)) and MassBank (https://massbank.eu/MassBank/ (accessed on 13 March 2024)). Only features with more than 50% nonzero measurement values in at least one group were retained for further analyses. In addition, the sweet–bitterness-related metabolites were identified through the screening process conducted by BitterDB (https://bitterdb.agri.huji.ac.il/dbbitter.php (accessed on 9 March 2024)) and VirtualTaste (https://insilico-cyp.charite.de/VirtualTaste (accessed on 13 March 2024)). Based on the predicted results, core taste metabolites were selected for molecular docking analysis.

### 2.6. Computational Study of Sweet/Bitter Compound Binding to T1R2/T1R3 and T2R4/T2R14 Receptors

The core sweet–bitterness-related differentially expressed metabolites were selected. Molecular docking analysis was used to clarify key sweet/bitter/bittersweet substances using a T1R2/T1R3 model and a T2R4/T2R14 model. Homology modeling was employed to generate the three-dimensional (3D) structures of human T1R2/T1R3 and T2R4/T2R14 receptors, as their structures were not available. The amino acid sequences of the sweet taste receptors hT1R2 and hT1R3 and the bitter taste receptors hT2R4 and hT2R14 were retrieved from the protein database (NCBI, http://www.ncbi.nlm.nih.gov/protein/ (accessed on 13 June 2024)) with accession numbers NP_689418.2, NP_689414.1, NP_058640.1, and NP_076411 [28]. These sequences were then submitted to the I-TASSER online modeling server, which utilized the LOMETS multi-threaded method to identify structural templates in the PDB database. Using protein sequences, 3D structures were constructed and evaluated for quality using ERRAT, Ramachandran plots, and C-scores. The best model was selected for molecular docking, with the DoGSiteScorer website (https://proteins.plus/ (accessed on 13 March 2024)) predicting docking sites for the receptor models [30]. Sweet /bitter/bittersweet compounds were docked into the predicted sites using AutoDock Vina, generating 20 poses for each ligand to interact with the protein binding site [31]. The binding energy (Affinity) of the ligand conformation was a key parameter, with lower binding energy indicating a more stable conformation and stronger binding affinity.

### 2.7. Statistical Analysis

The taste attributes obtained via electronic tongue analyses are expressed as means ± standard deviation and were subjected to one-way analysis of variance (ANOVA) with Duncan’s test using SPSS (25.0, IBM) software. Hierarchical clustering analysis was performed using TBtools software (TBtools, Guangzhou, China), while SIMCA-P 14.1 multivariate statistical software (Umetrics, Umea, Sweden) was used for generating radar plots, principal component analysis (PCA), and orthogonal partial least squares discriminant analysis (OPLS-DA) along with other visual representations. The molecular docking results were visualized using PyMOL 2.2.0 software (PyMOL, DeLano Scientific LLC).

## 3. Results and Discussion

### 3.1. Electronic Tongue Analysis and Correlation Analysis 

**Figure 1:**
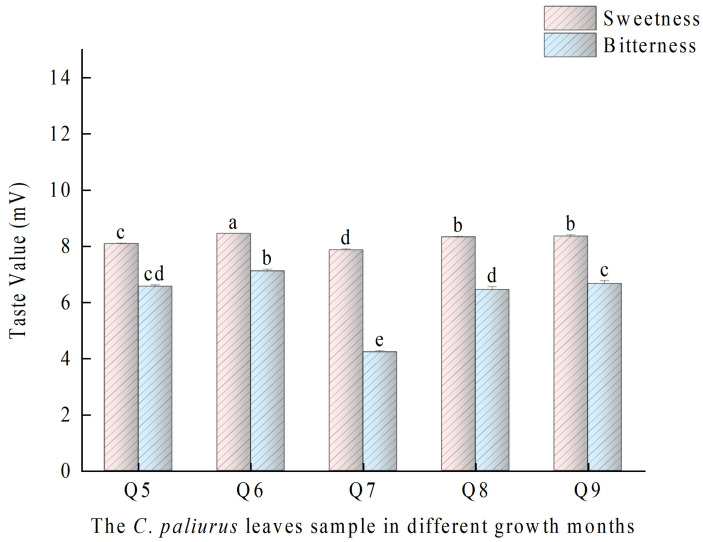
Bar chart of sweetness and bitterness values of *C. paliurus* leaves in different growth months. Notes: Q5–Q9 refer to the samples of the leaves of *C. paliurus* collected from May to September. ^a–e^ Means within the same taste value with different superscripts differ significantly (*p* < 0.05).

The taste attributes of the *C. paliurus* leaves in the five different months were measured by the electronic tongue system, as shown in Table 1. The tasteless points for all indices were set at 0, and values exceeding these tasteless points were deemed meaningful [32,33]. The taste values of sweetness, bitterness, and umami all had values higher than the tasteless points (*p* <0.05), signifying that these were the effective sensory indices for *C. paliurus* leaves in different months. As an herbal tea, *C. paliurus* tea has gradually entered the field of view of more consumers, but the widespread coexistence of sweetness and bitterness observed in the taste attributes, which cannot meet the high-quality taste expectations of consumers, has resulted in low market recognition of *C. paliurus* tea products. Therefore, this study primarily focused on the sweetness and bitterness during the five different growth months of *C. paliurus* leaves. As illustrated in Figure 1, the sweetness of *C. paliurus* leaves harvested in July was slightly lower than that in other months, meanwhile, the bitterness was significantly lower than that in other months (*p* < 0.5). According to previous reports [34], sweetness and bitterness of herbal teas were mostly determined by chemical composition, such as flavonoids, polyphenols, polysaccharides, and saponins. Hence, further main active compounds analysis and nontargeted metabolomics analysis of the five different growth months of *C. paliurus* leaves were carried out.

### 3.2. Changes in TPC, TFC, TP and TSC of C. Paliurus Leaves in Different Growth Periods

**Figure 2:**
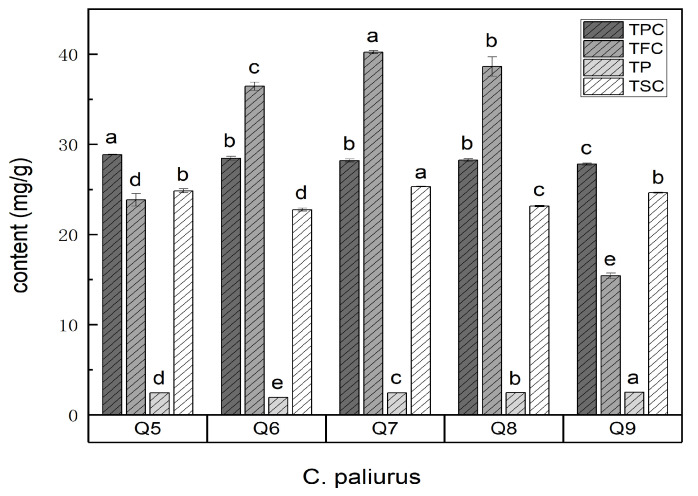
TPC, TFC, TP, and TSC from *C. paliurus* leaves. Notes: TPC (total phenolic content), TFC (total flavonoid content), TP (total polysaccharide content), and TSC (total saponin content). Q5–Q9 refer to the samples of the leaves of *C. paliurus* collected from May to September. ^a–e^ Means within the same indicator with different superscripts differ significantly (*p* < 0.05).

**Figure 3:**
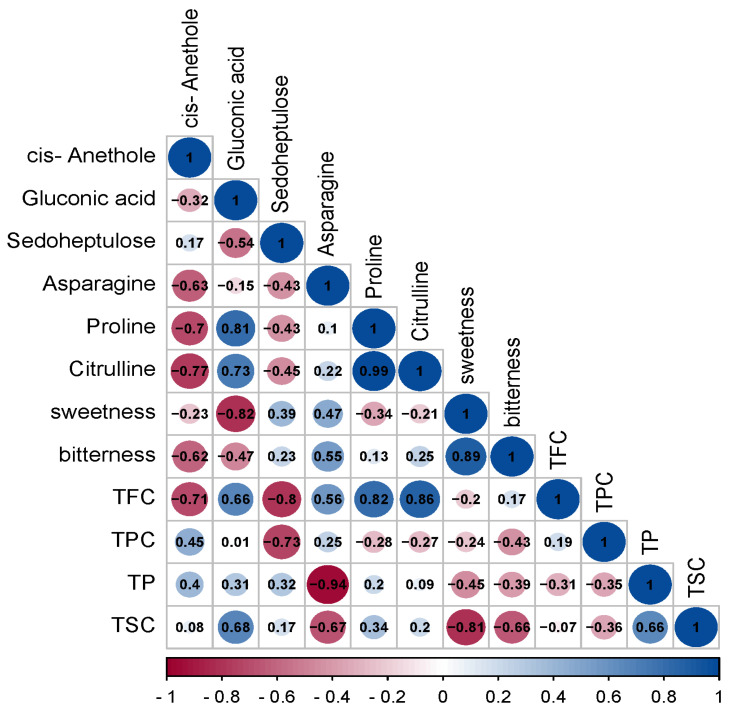
Associations between six key metabolites and the rest of the indices. Notes: The color gradient from red to blue on the chart illustrates the intensity of correlation coefficients, with red indicating the weakest association and blue representing the strongest.

Flavonoids, polyphenols, polysaccharides, and saponins were widely present in the *C. paliurus* leaves, which were harvested in the subtropical monsoon climate of Huping Mountain, Changde, Hunan Province [35,36]. The TPC, TFC, TP, and TSC of the *C. paliurus* leaves in the different growth months are presented in Figure 2. There was no significant difference in the TPC of the *C. paliurus* leaves in the different growth months (*p* < 0.05), suggesting that the phenolic levels may not have been significantly influenced by the progression of the growth period, or that other factors, such as regional climate conditions, might have played a more dominant role in modulating the TPC. The TFC could have been linked to increased solar radiation and temperatures in the early growth months, promoting synthesis of flavonoids, followed by decline as the season progressed and temperatures began to moderate. In terms of the TP and TSC, a reversal was observed in the TP of the *C. paliurus* leaves in the different growth periods, where an initial decline was succeeded by an upward movement, indicating a potential turning point or recovery. This pattern might have been related to the plants’ adaptation to climatic conditions, such as increased rainfall in the early growth months providing sufficient hydration for polysaccharide synthesis, followed by a period of adjustment as the climate became hotter and drier. Moreover, the TSC of the *C. paliurus* leaves displayed periodic fluctuations, which could have been associated with seasonal factors. The periodic nature of these fluctuations might have been a response to changing environmental conditions, including variations in temperature, rainfall, and humidity throughout the growth months. In fact, phenolic acids are responsible for *C. paliurus* tea’s distinctive color and taste, and bioactive components contribute to its antibacterial, antiviral, antioxidation, antihypertension, and hypolipidemic activities [3,37,38]. Previous studies on chemical composition showed that *C. paliurus* leaves contain rich phenolic acid compounds, especially flavonoids [4,39,40]. As shown in Figure 2, the active components of the *C. paliurus* exhibited dynamic seasonal variations, and the highest TFC, TP, and TSC were found in the *C. paliurus* leaves harvested in July and could be attributed to the climatic conditions of July. In fact, July is in the middle of summer compared to other months, and generally Huping Mountain experiences higher temperatures, higher humidity, and more variable rainfall patterns in July compared to other months. These conditions could favor biosynthesis and accumulation of secondary metabolites such as flavonoids, polysaccharides, and saponins in *C. paliurus* leaves. However, there was no significant correlation between the sweetness and bitterness of the *C. paliurus* leaves and the TPC, TFC, TP and TSC (Figure 3). Nevertheless, previous research has suggested that a higher polyphenol content contributes to increased bitterness [34]. The bitterness induced by polyphenols is influenced not only by their overall content but also by the specific composition of the polyphenols, their bitterness thresholds, and the effective concentrations at which they are present. Furthermore, empirical evidence has suggested that perceived intensity of bitterness does not exhibit a direct linear correlation with concentration of these compounds [41]. Consequently, nontargeted metabolomics and molecular docking technology were employed to further analyze the intrinsic relationship between the sweet /bitter/bittersweet substances.

### 3.3. Nontargeted Metabolomics Analysis of C. Paliurus Leaves

**Figure 4:**
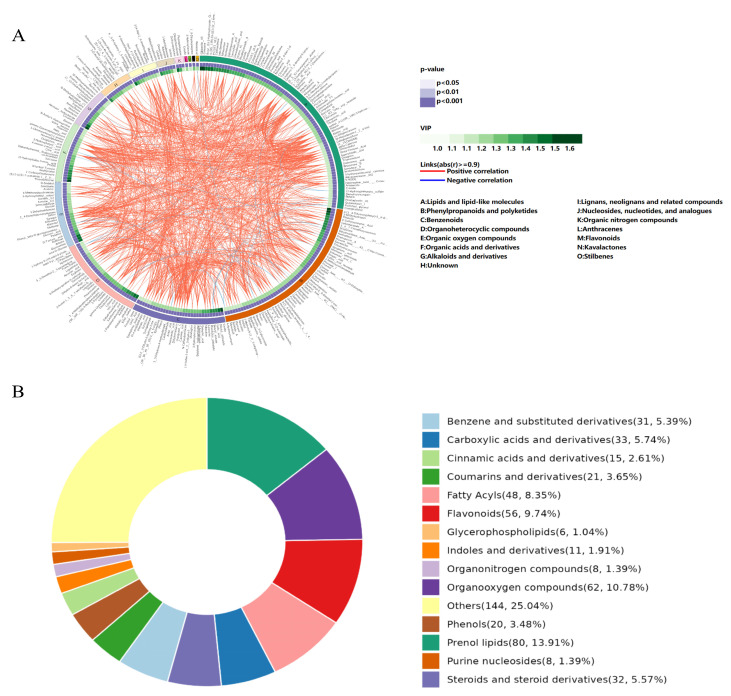
Nontargeted metabolomics analysis. (**A**) The circus plot of differential metabolites. Notes: Circus circle plot is arranged from the outside inwards in the following order: metabolite names, HMDB classifications of metabolites, p-values, VIP from PLS-DA analysis, and correlation lines. Arcs connecting any two points on the circle are called chords, indicating a correlation between the two points. (**B**) The proportion of various metabolites in *C. paliurus* leaf samples. Notes: Various color-coded sections denote items categorized under distinct chemical groups, with the corresponding percentages indicating the proportion of items within each chemical category. The proportion of metabolites is calculated as a fraction of the total metabolites detected. Those metabolites lacking a defined chemical classification are categorized as “others”.

Initial investigation revealed that *C. paliurus* leaves possess notable bitterness in addition to their sweetness, factors that significantly affect their sensory quality. In this study, nontargeted metabolomics based on UHPLC-MS/MS analysis was undertaken to identify the potential sweet and bitter components within the *C. paliurus* leaves. Utilizing the PRIMe database (PRIMe: Platform for RIKEN Metabolomics) for search and metabolite analysis, a total of 1571 secondary metabolites were successfully characterized from the *C. paliurus* leaves, with both positive and negative patterns detected. A circus plot was used to analyze the associations between the differential metabolites and for classification. Metabolite name, HMDB classification of metabolite, *p*-value, VIP from OPLS-DA analysis, and correlation line were listed from outside to inside. As shown in Figure 4A, these different metabolites included 603 positive correlations (38.4%) and 441 negative correlations (28.1%). Of these, 575 differential metabolites were identified and systematically categorized into 15 distinct classes (Figure 4B). These categories included 80 prenol lipids (13.91%), 62 organooxygen compounds (10.78%), 56 flavonoids (9.74%), 48 fatty acyls (8.35%), 33 carboxylic acids and derivatives (5.74%), 32 steroids and steroid derivatives (5.57%), 31 benzene and substituted derivatives (5.39%), 21 coumarins and derivatives (3.65%), 20 phenols (3.48%), 15 cinnamic acids and derivatives (2.61%), 11 indoles and derivatives (1.91%), 8 organonitrogen compounds (1.39%), 8 purine nucleosides (1.39%), 6 glycerophospholipids (1.04%), and 144 undefined compounds (25.04%). 

**Figure 5:**
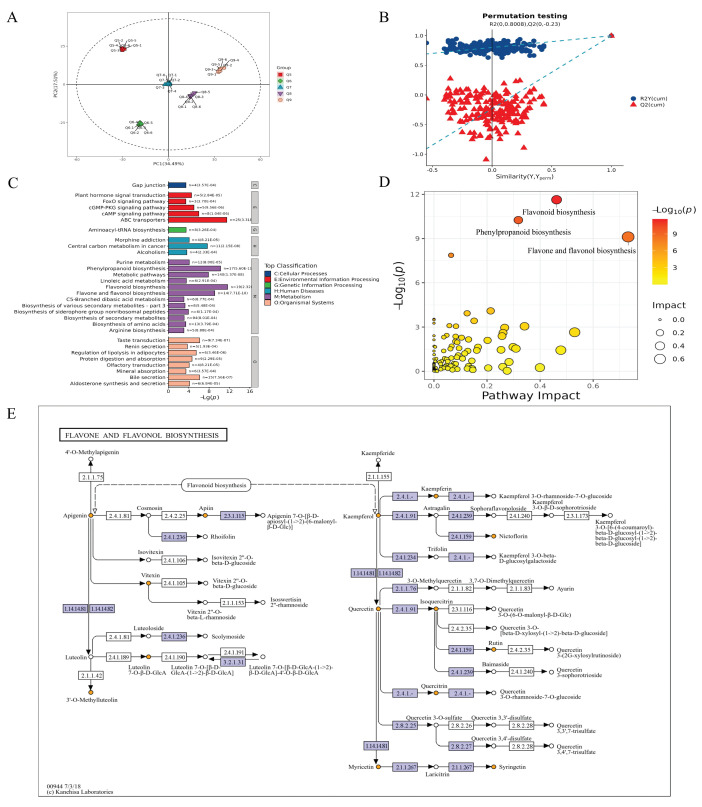
Multivariate analyses of *C. paliurus* leaf samples. (**A**) Principal component analysis (PCA) of metabolite profiles across different groups. (**B**) OPLS-DA permutation test plot of the comparison groups. (**C**) The bar chart of significant KEGG enrichment pathways. (**D**) The diagram of pathway impact. (**E**) The flavone and flavanol biosynthesis pathways.

The principal component analysis (PCA), partial least squares discriminant analysis (PLS-DA), and orthogonal projections to latent structures discriminant analysis (OPLS-DA) have been used to pinpoint the metabolite combinations that explain the highest variance and to illustrate the clustering patterns of the tea samples [42]. All the metabolites were subjected to multivariate analysis using SIMCA-P 14.1 multivariate statistical software. The results of the PCA (Figure 5A) illustrated that PC1 and PC2 accounted for 34.49% and 17.51% of the total variation, respectively. In addition, five samples were completely separated in the PCA map, which indicated that there were significant differences in the metabolite content of samples from different growth months. The OPLS-DA utilizes a predictive principal component (t1) for group difference detection and multiple orthogonal components for intra-group variability. Therefore, to obtain a higher level of population separation and better understand the differences between the *C. paliurus* leaves in different months, the OPLS-DA was used for classification (Figure 5B). Permutation test cross-validation confirmed the robustness of the OPLS-DA model, exhibiting R2 and Q2 intercepts of 0.999 and 0.9975, which proved that the model had good prediction ability (Table S2). 

Since different metabolites coordinate their biological functions, the Kyoto Encyclopedia of Genes and Genomes (KEGG) pathway-based analysis is instrumental in further elucidating their roles [43]. Fisher’s exact test was used to analyze and calculate the significance level of the metabolite enrichment in each pathway to identify the metabolic and signal transduction pathways that were significantly affected. The KEGG enrichment pathway map is presented in Figure 5C. The x-axis represents the negative logarithm of the *p*-values, while the y-axis indicates the specific pathways. Colors of the bars were assigned based on the KEGG pathway level 1 classifications. 

As shown in Figure 5D, the majority of the differentially expressed metabolites were primarily associated with biosynthetic pathways, such as flavonoid and flavanol biosynthesis, flavonoid biosynthesis, phenylpropanoid biosynthesis, and other pathways highlighted by the KEGG enrichment analysis, all of which exhibited statistical significance(*p* < 0.05). As the most significant pathways, the flavone and flavanol biosynthesis pathways have been extensively studied in *C. paliurus* [44]. Given the visual representation of the pathways in Figure 5E, the complex network of synthesis and transformation of key flavonoid components such as apigenin, luteolin, and their glycosides and derivatives is revealed. The diagram captures the interplay between these compounds and their conversions into various forms. Additionally, it highlights synthesis and transformation of three flavanol components: kaempferol, quercetin, and myricetin, as well as their derivatives. Flavanols belong to the class of flavonoids, which mainly exist as glycosides in *C. paliurus* leaves, and have a certain contribution to the taste quality of tea, especially bitterness, that has been noted in other herbal teas [45]. In this study, the data of the nontargeted metabolomics analysis showed a correlation with the taste indices of the electronic tongue analysis, in which luteolin (VIP = 1.22) and apigenin (VIP = 1.14) were the core contributions to the difference between these *C. paliurus* leaf samples as indicated by high VIP values, which were responsible for the tea infusion’s bitterness [45]. During the growth of the *C. paliurus* leaves in the different months, the concentration of luteolin exhibited a pattern of initial increase peaking in June, followed by a decrease. In contrast, the concentration of apigenin followed an inverse trend, initially declining and then rising, with the lowest concentration observed in June. Overall, the core bitterness-related metabolic (luteolin and apigenin) variation between the *C. paliurus* leaves’ growth stages was revealed via nontargeted metabolomics analysis. However, *C. paliurus* leaves have an observed widespread coexistence of sweetness and bitterness in the taste attributes, and key metabolites of the potential “sweet-bitterness” in *C. paliurus* leaves have not been reported. Therefore, a deeper investigation into the differential metabolites that contribute to the characteristic sweet-bitterness of *C. paliurus* leaves was carried out. 

To identify the differential metabolites that may be related to the sweet/bitter taste of *C. paliurus*, the 575 different metabolites were further screened by BitterDB (https://bitterdb.agri.huji.ac.il/dbbitter.php (accessed on 9 March 2024)) and the related literature and using VirtualTaste (https://insilico-cyp.charite.de/VirtualTaste (accessed on 13 June 2024)) to predict the sweet and bitter taste. A total of 68 metabolites were predicted to be active in sweetness. The Variable Importance for the Projection (VIP) score measures metabolite expression patterns’ impact on sample classification, identifying the key discriminative. Therefore, the core metabolites with VIP ≥ 1 were selected from VirtualTaste and were used to predict important explanatory variables for sweet and bitter substances. Detailed information about these metabolites is provided in Table S1. These metabolites included 20 phenylpropanoids and polyketides (29.0%), 17 lipids and lipid-like molecules (24.6%), 12 organic oxygen compounds (17.4%), 8 organic acids and derivatives (11.6%), 6 benzenoids (8.7%), 2 lignans, neolignanes, or related compounds (2.9%), 1 kavalactone (1.4%), 1 organic nitrogen compound (1.4%), 1 organoheterocyclic compound (1.4%), and 1 superclass (1.4%).

### 3.4. Molecular Docking of Candidate Sweet/Bitter Substances with the T1R1/ T1R3 and T2R4/T2R14 Receptors

To investigate the potential sweet/bitter/bittersweet substances, molecular docking analysis was conducted to evaluate the stability of and the interactions between these substances and the human sweet receptors (T1R1/TIR3) and bitter receptors (T2R4/T2R14). The 68 core sweetness metabolites predicted as active in sweetness were further screened (Sweet Prediction > 0.7, Bitter Prediction > 0.7) and categorized into four groups for detailed analysis: Category 1 included the bitter substances identified in BitterDB with active Bitter Predictions in VirtualTaste; Category 2 comprised the bitter substances listed in BitterDB but predicted as inactive in VirtualTaste; Category 3 encompassed the non-bitter substances according to BitterDB with active Bitter Predictions in VirtualTaste; Category 4 consisted of the bitter substances found in BitterDB but rated as inactive in VirtualTaste (Table S2).

**Figure 6:**
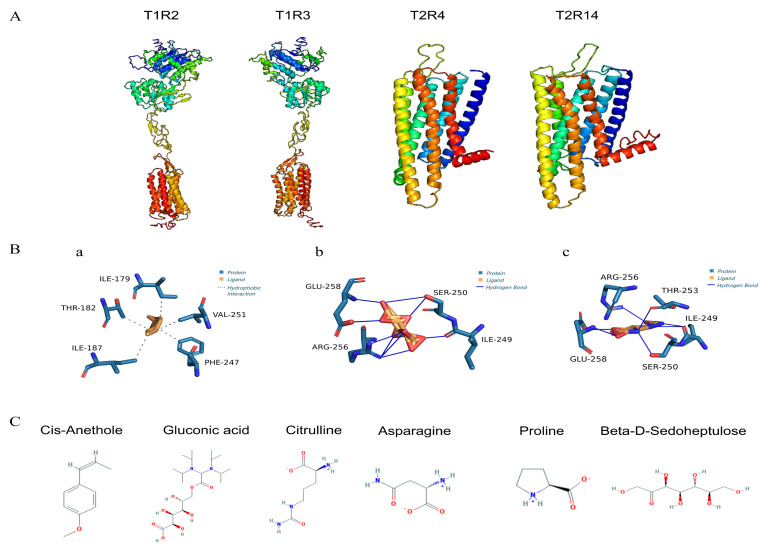
Homologous modeling and screening process of molecular docking. (**A**) Sweet receptors (T1R1/TIR3) and bitter receptors (T2R4/T2R14). (**B**) Interaction patterns of hT2R14 with cis-anethole, gluconic acid and citrulline; (**a**) hT2R14–cis-anethole, (**b**) hT2R14–gluconic acid, (**c**) hT2R14–citrulline. (**C**) Six core sweet- and bitter-related metabolites.

The amino acid sequences of the sweet taste receptors hT1R2 and hT1R3 and the bitter taste receptors hT2R4 and hT2R14 (IDs NP_689418.2, NP_689414.1, NP_058640.1, NP_076411.1, respectively) were downloaded from the NCBI database (Figure S1). Each sequence was submitted individually to the I-TASSER online modeling server, which employs the LOMETS multi-threaded method to identify structural templates in the PDB database. The server selected the top-scoring template from each of the 10 threaded programs based on Z-score, a measure of template alignment quality. Following template selection, I-TASSER constructed a full-length protein model by assembling contiguous segments derived from the threaded alignment through a Monte Carlo simulation that replicated an exchange process. This computational approach generated comprehensive 3D protein models suitable for further analysis and molecular docking studies [46]. The quality of the resulting models was assessed using ERRAT, Ramachandran plots, and C-score to select the optimal model for the subsequent molecular docking (Figure 6A).

The DoGSiteScorer (https://proteins.plus/ (accessed on 13 June 2024)) website was utilized to predict docking sites for the hT1R2, hT1R3, hT2R4, and hT2R14 proteins. Sweet taste receptor binding sites (for hT1R2 and hT1R3) were identified in the Venus Flytrap Module (VFTM), situated in the central cleft between the two larger lobes. In contrast, bitter taste receptor binding sites (for hT2R4 and hT2R14) were located in the central cavity formed by seven transmembrane helices, consistent with the prior research on docking sites of the sweet and bitter taste receptors with various ligands [47,48].

The small molecules, after undergoing pre-treatment, were subjected to docking simulations with the four pre-processed receptor models using the Vina software 1.2.0. The binding energy (Affinity) of the ligand conformation is defined as a key parameter in the process of molecular docking, where a lower binding energy indicates a more stable ligand conformation. This stability correlates with a stronger binding affinity to the protein receptor, suggesting a potentially higher inhibitory effect on the receptor’s activity [49]. Given the direct correlation between the sweetness of a ligand and its interaction energy with sweetness receptors, hT1R2 and hT1R3 are capable of independently yet synergistically generating sweetness signals. This dual capacity facilitates a cumulative effect, wherein the combined action of these receptors can significantly amplify the perceived sweetness [50]. To elucidate the differential effects on taste perception, an analysis comparing the binding affinities of the sweet and bitter taste receptors was conducted. Specifically, this study calculated the sum of the lowest binding energies (denoted as As) for the binding sites of the sweet taste receptors hT1R2 and hT1R3. At the same time, the sum of the lowest binding energies of the bitter receptors hT2R4 and hT2R14 (expressed as Ab) was also predicted. This comparative approach allowed us to assess the relative inhibitory potential of the ligands on the receptors responsible for sweet and bitter taste sensations. The relevant docking results are shown in Table 2. The data of correlation analysis pointed out a positive correlation between the sum of the lowest binding energies of the sweet receptor (As) and the Sweet Predict scores, indicating that the likelihood of the ligand being perceived as sweet increased with an increase in As. Conversely, a negative correlation was observed between the sum of the lowest binding energies for the bitter taste receptors (Ab) and the Bitter Predict scores, indicating that higher Ab values were linked to a reduced likelihood of bitterness perception. Thus, As ≥ −15 and Ab ≥ −15 were identified as thresholds for the identification of high sweetness and low bitterness metabolites. Finally, six compounds (cis-anethole, gluconic acid, beta-D-Sedoheptulose, asparagine, proline, citrulline) were selected to match the conditions (As ≥ −15 and Ab ≥ −15) (Figure 6C). Among them, cis-anethole belongs to benzenoids; gluconic acid and beta-D-Sedoheptulose belong to organic oxygen compounds. Asparagine, proline, and citrulline belong to organic acids and derivatives. Based on the average standardized peak area data from Table 3, the levels of the six metabolites (cis-anethole, gluconic acid, beta-D-Sedoheptulose, asparagine, proline, and citrulline) in the *C. paliurus* leaves remained high from May to September. Sedoheptulose exhibited the highest content, with significant fluctuations across different months. In contrast, the levels of the other five metabolites were relatively consistent during June, July, and August. Therefore, these six core metabolites with high sweetness and low bitterness could be potential candidates for taste modulation in *C. paliurus* leaves. These findings suggest that by selectively enriching these compounds or by employing targeted breeding and genetic modification strategies, it may be possible to enhance the palatability of *C. paliurus* products. Moreover, this method enabled the targeted identification of compounds that were likely contributors to the sweetness and bitterness, thereby deepening our comprehension of the molecular underpinnings of taste perception within this specific context. 

Currently, the hT2R14 receptor has been recognized as one of the most broadly responsive bitterness receptors, capable of detecting a wide array of bitter compounds [51,52]. Potency of these bitter substances can be significantly mitigated through inhibition of the hT2R14 receptor, offering a potential strategy for modulating bitterness intensity in various applications. Thr (threonine), Asp (aspartic acid), and Phe (phenylalanine) have been identified as the key amino acids responsible for docking of bitter taste blockers with hT2R14 in molecular docking calculations [53]. The interactions between the hT2R14 receptor and cis-anethole, gluconic acid, and citrulline were assessed using the PLIP web service (Protein-Ligand Interaction Profiler, https://projects.biotec.tu-dresden.de/plip-web/plip/index (accessed on 11 June 2024)), which provides an online platform for predicting and analyzing protein–ligand interactions. The results of this prediction were then imported into PyMOL, a molecular visualization tool, to render three-dimensional representations of the complexes. According to Figure 6B, the analyses of the ligands’ interactions with the hT2R14 model at the docking site revealed distinct binding characteristics for the core metabolites from the *C. paliurus* leaves. Cis-anethole demonstrated no interaction with Asp but formed hydrogen bonds with Thr-182 and Phe-247. Citrulline was found to engage in hydrophobic interactions with Thr-253. In contrast, gluconic acid did not create any significant bonds with amino acids Thr, Asp, or Phe. The differential binding profiles of these metabolites to the hT2R14 receptor were reflected in their respective binding energies (Ab), with cis-anethole showing the lowest, followed by citrulline, with gluconic acid exhibiting the highest. This order of binding energies (cis-anethole<citrulline<gluconic acid) suggests a potential inverse relationship between the binding strength to the receptor and the perceived bitterness of the metabolites. This methodological approach, therefore, offers a promising strategy for predicting bitterness intensity of differential metabolites in *C. paliurus* leaves. By identifying metabolites that exhibit weaker binding to the hT2R14 receptor, it may be possible to select or modify compounds that contribute favorably to the taste profile of *C. paliurus* tea, enhancing palatability and potential health benefits. Furthermore, the identification of key amino acids involved in the binding of bitter compounds to the hT2R14 receptor opens up avenues for developing taste blockers or modifiers that can reduce the bitterness of *C. paliurus* leaves. This could involve the design of small molecules that can competitively inhibit binding of bitter compounds to the receptor, thereby reducing perceived bitterness.

To statistically calculate the relationships between the core six metabolites and the taste intensity, Spearman’s correlation analysis coefficient was utilized in Figure 6. There was a significant correlation between the sensory characteristics (sweetness and bitterness) and the six core metabolites. The absolute value of a correlation coefficient in statistics indicates the strength of the correlation: the closer the coefficient is to 1 or −1, where −1 means completely negative correlation, 1 means completely positive correlation, and 0 means completely irrelevant, the stronger the correlation. [54]. According to Figure 6, asparagine exhibited a very strong negative correlation with the TP (−0.94), sedoheptulose showed a very strong negative correlation with the TFC (−0.8), and cis-anethole had a strong negative correlation with the TFC (−0.71). Gluconic acid demonstrated a strong positive correlation with both the TSC (0.68) and TFC (0.66), while asparagine had a strong negative correlation with the TSC (−0.67). These correlations hinted at a fascinating interaction between the metabolite profiles and the taste profiles of the *C. paliurus* leaves. It was reasonable to deduce that the concentrations of cis-anethole and sedoheptulose could significantly influence the bitterness intensity of the *C. paliurus* leaf samples, considering their direct associations with the TFC and TPC, respectively. In fact, bitterness was generally considered undesirable; still, they were important for providing the complex sensory perceptions of *C. paliurus* teas. Therefore, it is possible to design small molecule compounds that can competitively inhibit the binding of bitter compounds such as cis-anethole and sedoheptulose to receptors, thereby reducing the perceived bitterness. Meanwhile, asparagine had high Pearson correlation coefficients with bitterness and sweetness and negative correlation with the TSC and TP; the concentration of asparagine in the *C. paliurus* leaves was likely a significant determinant of the sweetness intensity. However, as one of the components of the synthetic sweetener aspartame, L-asparagine was without taste, while D-asparagine was intensely sweet. Details and mechanisms of interactions between chiral sweet molecules and sweet receptors remain to be elucidated [55]. Therefore, this study advanced our understanding of the metabolic changes and the sweetness and bitterness taste in the different growth months of the *C. paliurus* leaves and provided a new method for detailing the interactions between sweet molecules and sweet receptors.

Moreover, growing demand for tasty and healthy food has driven the development of low-calorie sweeteners originated from natural sources [56]. Natural sweeteners generally refer to a kind of sweet chemical component directly extracted from nature or properly modified, mainly secondary metabolites of plants, which include steviol glycosides, rebaudiosides, Luo Han Guo (monk fruit), etc. [57]. Natural sweeteners such as stevia have been widely used in high-end beverages, food, and health products and have been recognized by consumers, with an increasing position in the international context of sugar reduction [58]. Of note, leaves of the stevia plant have been widely used to make a sugar substitute called stevia. However, stevia has a bitter aftertaste, which makes it similar to the leaves of *C. paliurus*. Therefore, the leaves of *C. paliurus* have potential application value for developing natural sweeteners. This study provided a theoretical basis and method for design and development of a *C. paliurus* natural sweetener and related products.

## 4. Conclusions

This study explored the differential metabolites contributing to the sweet and bitter taste in *C. paliurus* leaves from different growth months using a multiplatform metabolomics approach, including UHPLC-MS/MS, electronic tongue assay, and molecular docking approaches. The results indicated significant variations in the taste characteristics and the content of bioactive substances, with leaves harvested in July exhibiting relatively high sweetness and the lowest bitterness. Then nontargeted metabolomics revealed 1571 differential metabolites, with 575 identified as key metabolites based on VIP values. Molecular docking analysis elucidated the interactions between these metabolites and the sweetness (hT1R2/hT1R3) and bitterness (hT2R4/hT2R14) receptors, revealing a positive correlation between the binding affinities and perceived sweetness, and a negative correlation for bitterness. Notably, six core metabolites (cis-anethole, gluconic acid, beta-D-Sedoheptulose, asparagine, proline, and citrulline) were identified as potentially modulating the sweet–bitter taste of the *C. paliurus* leaves. This discovery highlighted the effectiveness of the integrated analytical and in-computer simulation screening method in uncovering and characterizing compounds critical to taste profiles of food products. Moreover, this study provided a foundation for enhancing the taste attributes of *C. paliurus* leaves, which could increase their marketability and meet consumer demands for high-quality taste experiences. Further studies are ongoing in the author’s lab, focusing on the formation mechanisms of the key sweet–bitterness related components in *C. paliurus* leaves. We hope to report more about these advancements in the future.

## Figures and Tables

**Table 1:** Determination of taste characteristics by electronic tongue of *C. paliurus* harvested in different months.

Taste Characteristic	Samples of *C. paliurus*				
	Q5	Q6	Q7	Q8	Q9
Sweetness	8.09 ± 0.02 ^c^	8.45 ± 0.00 ^a^	7.86 ± 0.04 ^d^	8.33 ± 0.01 ^b^	8.36 ± 0.04 ^b^
Sourness	−24.20 ± 0.15 ^c^	−26.25 ± 0.06 ^e^	−22.77 ± 0.23 ^a^	−24.55 ± 0.11 ^d^	−23.12 ± 0.13 ^b^
Bitterness	6.57 ± 0.05 ^cd^	7.12 ± 0.06 ^b^	4.23 ± 0.04 ^e^	6.46 ± 0.1 ^d^	6.67 ± 0.10 ^c^
Astringency	3.10 ± 0.23 ^a^	2.21 ± 0.24 ^c^	2.46 ± 0.16 ^bc^	0.67 ± 0.31 ^e^	1.24 ± 0.24 ^d^
Bitter Aftertaste	2.26 ± 0.06 ^c^	2.09 ± 0.05 ^d^	1.59 ± 0.04 ^f^	1.85 ± 0.05 ^e^	2.40 ± 0.08 ^b^
Astringency Aftertaste	2.76 ± 0.15 ^a^	2.13 ± 0.11 ^b^	2.74 ± 0.16 ^a^	1.39 ± 0.09 ^d^	1.70 ± 0.11 ^c^
Umami	6.35 ± 0.19 ^b^	7.90 ± 0.17 ^a^	4.65 ± 0.21 ^d^	5.55 ± 0.19 ^c^	3.98 ± 0.20 ^e^
Richness	2.48 ± 0.23 ^b^	2.08 ± 0.14 ^c^	2.33 ± 0.12 ^bc^	1.50 ± 0.07 ^d^	1.56 ± 0.19 ^d^

Notes: Standard error of means (n = 3). ^a–f^ Means within the same row with different superscripts differ significantly (*p* < 0.05). Q5–Q9 refer to the samples of the leaves of *C. paliurus* collected from May to September, 2023.

**Table 2:** Relevant metabolites screened for molecular docking.

Metabolite Name	hT1R2	hT1R3	hT2R4	hT2R14	As	Ab
Category 1						
Ganoderic Acid B	−13.0	−11.9	−12.3	−11.7	−24.9	−24.0
Deoxycholic acid	−10.9	−10.8	−11.7	−10.7	−21.7	−22.4
Indole-3-acetyl-valine	−9.2	−8.9	−8.6	−7.9	−18.1	−16.5
Category 2						
Ginsenoside Rf	−10.1	−12.7	−10.6	−10.4	−22.8	−21.0
FA 18:3;O	−6.8	−8.7	−7.9	−7.0	−15.5	−14.9
cis-Anethole	−5.8	−5.6	−5.8	−5.6	−11.4	−11.4
Icariside F2	−10.1	−9.7	−8.4	−9.0	−19.8	−17.4
Forsythoside E	−10.3	−10.7	−8.0	−8.7	−21.0	−16.7
Gluconic acid	−5.7	−5.6	−5.0	−5.1	−11.3	−10.1
Olivil 4’-O-Glucoside	−9.2	−10.6	−9.2	−8.7	−19.8	−17.9
Category 3						
Centaurein	−10.5	−10.0	−8.4	−9.1	−20.5	−17.5
Iridin	−9.2	−10.0	−8.8	−8.0	−19.2	−16.8
Syringetin	−9.9	−9.6	−8.3	−8.3	−19.5	−16.6
Dihydromelilotoside	−8.6	−8.5	−7.8	−7.1	−17.1	−14.9
Lamiide	−10.3	−10.1	−8.6	−8.0	−20.4	−16.6
Category 4						
Sedoheptulose	−7.0	−6.8	−5.8	−6.6	−13.8	−12.4
Hematoxylin	−10.5	−9.3	−9.4	−8.9	−19.8	−18.3
Asparagine	−5.1	−5.4	−4.6	−5.2	−10.5	−9.8
Proline	−5.2	−5.3	−5.0	−5.4	−10.5	−10.4
Citrulline	−5.7	−5.8	−5.1	−5.7	−11.5	−10.8
Gaultherin	−9.6	−9.0	−7.8	−8.1	−18.6	−15.9
Apiopaeonoside	−9.7	−9.3	−8.5	−8.2	−19.0	−16.7

Notes: the sum of the lowest binding energies for the sweet taste receptors (As); the sum of the lowest binding energies for the bitter taste receptors (Ab).

**Table 3:** The mean values of the normalized peak areas of core compounds of *C. paliurus* leaves.

Metabolite Name	Mean Value of Normalized Peak Area				
	Q5	Q6	Q7	Q8	Q9
cis-Anethole	2.19 × 10^8 d^	2.63 × 10^8 c^	3.68 × 10^8 a^	3.57 × 10^8 a^	3.15 × 10^8 b^
Gluconic acid	9.52 × 10^10 a^	1.11 × 10^10 c^	6.85 × 10^10 b^	1.86 × 10^10 c^	9.58 × 10^9 c^
Sedoheptulose	1.08 × 10^9 b^	2.15 × 10^9 b^	1.85 × 10^9 b^	2.26 × 10^9 b^	1.04 × 10^10 a^
Asparagine	1.90 × 10^8 b^	4.33 × 10^8 a^	3.22 × 10^7 c^	9.19 × 10^7 c^	1.51 × 10^7 d^
Proline	2.59 × 10^10 a^	3.70 × 10^9 c^	5.04 × 10^9 b^	5.75 × 10^9 b^	2.97 × 10^9 c^
Citrulline	3.59 × 10^8 a^	1.00 × 10^8 b^	6.53 × 10^7 c^	1.09 × 10^8 b^	5.82 × 10^7 c^

Notes: Q5–Q9 refer to the samples of the leaves of *C. paliurus* collected from May to September, 2023. ^a–d^ Means within the same row with different superscripts differ significantly (*p* < 0.05).

## Data Availability

The original contributions presented in the study are included in the article/Supplementary Material; further inquiries can be directed to the corresponding author.

## Supplementary Materials

The following supporting information can be downloaded at: https://www.mdpi.com/article/10.3390/foods13193089/s1, Figure S1: Template used for each receptor modeling; Table S1. The compound information for different growth months (Q5–Q9) detected in UHPLC-MS/MS analysis; Table S2. Molecular docking results of ligands with T1R2\T1R3 and T2R4\T2R14.
