# Supplementary material for: Metabolite Profiling and Identification of Sweet/Bitter Taste Compounds in the Growth of Cyclocarya Paliurus Leaves Using Multiplatform Metabolomics
Source: Foods. 2024 Sep 27;13(19):3089. doi: 10.3390/foods13193089 (PMC11475313; doi:10.3390/foods13193089)
Supplement: Supplementary file 1 [file foods-13-03089-s001.zip › foods-3176598-supplementary.pdf]

Supplementary Materials

Figure S1 Template used for each receptor modeling.

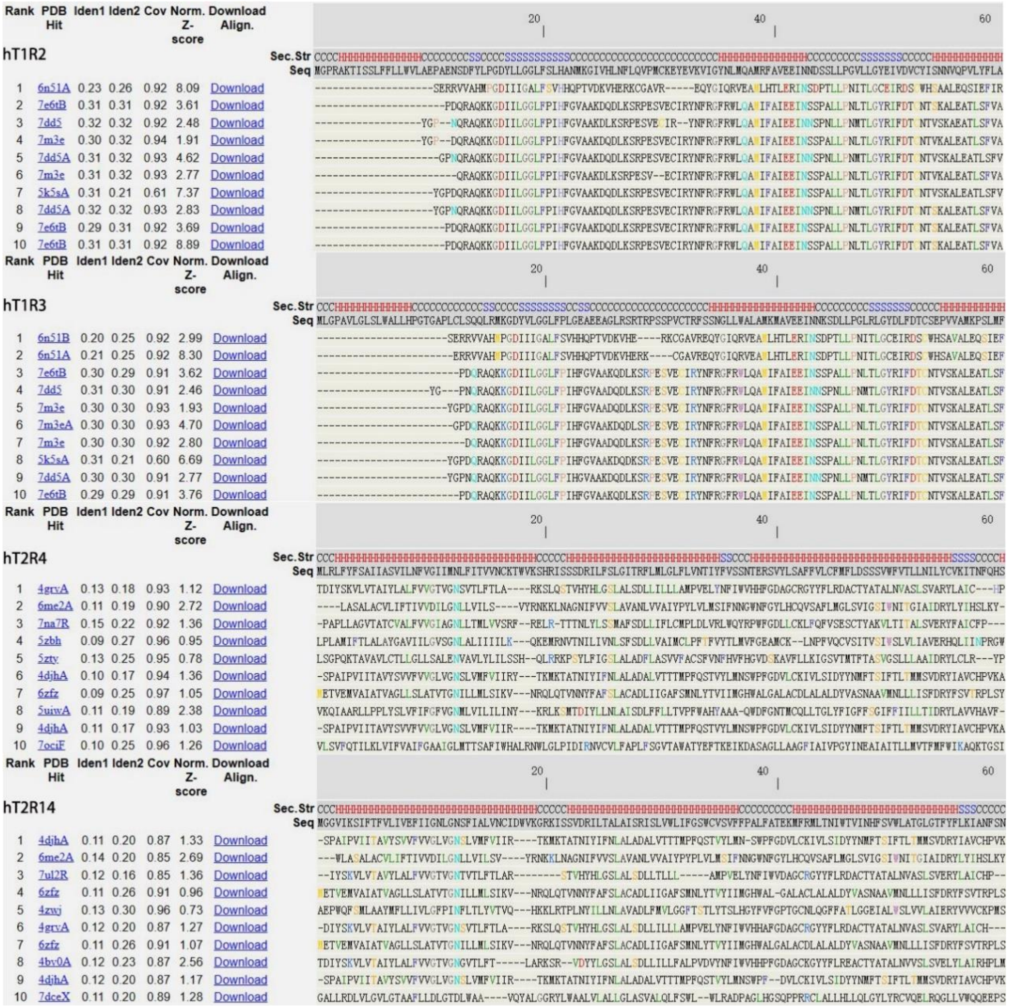

hT1R2

Rank	PDB Hit	Iden1	Iden2	Cov	Norm. Z-score	Download Align.
1	6n51A	0.23	0.26	0.92	8.09	Download
2	7e6tB	0.31	0.31	0.92	3.61	Download
3	7dd5	0.32	0.32	0.92	2.48	Download
4	7m3e	0.30	0.32	0.94	1.91	Download
5	7dd5A	0.31	0.32	0.93	4.62	Download
6	7m3e	0.31	0.32	0.93	2.77	Download
7	5k5sA	0.31	0.21	0.61	7.37	Download
8	7dd5A	0.32	0.32	0.93	2.83	Download
9	7e6tB	0.29	0.31	0.92	3.69	Download
10	7e6tB	0.31	0.31	0.92	8.89	Download

hT1R3

Rank	PDB Hit	Iden1	Iden2	Cov	Norm. Z-score	Download Align.
1	6n51B	0.20	0.25	0.92	2.99	Download
2	6n51A	0.21	0.25	0.92	8.30	Download
3	7e6tB	0.30	0.29	0.91	3.62	Download
4	7dd5	0.31	0.30	0.91	2.46	Download
5	7m3e	0.30	0.30	0.93	1.93	Download
6	7m3eA	0.30	0.30	0.93	4.70	Download
7	7m3e	0.30	0.30	0.92	2.80	Download
8	5k5sA	0.31	0.21	0.60	6.69	Download
9	7dd5A	0.30	0.30	0.91	2.77	Download
10	7e6tB	0.29	0.29	0.91	3.76	Download

hT2R4

Rank	PDB Hit	Iden1	Iden2	Cov	Norm. Z-score	Download Align.
1	4grvA	0.13	0.18	0.93	1.12	Download
2	6me2A	0.11	0.19	0.90	2.72	Download
3	7na7R	0.15	0.22	0.92	1.36	Download
4	5zbh	0.09	0.27	0.96	0.95	Download
5	5zty	0.13	0.25	0.95	0.78	Download
6	4djhA	0.10	0.17	0.94	1.36	Download
7	6zfz	0.09	0.25	0.97	1.05	Download
8	5uiwA	0.11	0.19	0.89	2.38	Download
9	4djhA	0.11	0.17	0.93	1.03	Download
10	7ociF	0.10	0.25	0.96	1.26	Download

hT2R14

Rank	PDB Hit	Iden1	Iden2	Cov	Norm. Z-score	Download Align.
1	4djhA	0.11	0.20	0.87	1.33	Download
2	6me2A	0.14	0.20	0.85	2.69	Download
3	7ul2R	0.12	0.16	0.85	1.36	Download
4	6zfz	0.11	0.26	0.91	0.96	Download
5	4zwj	0.13	0.30	0.96	0.73	Download
6	4grvA	0.12	0.20	0.87	1.27	Download
7	6zfz	0.11	0.26	0.91	1.07	Download
8	4bv0A	0.12	0.23	0.87	2.56	Download
9	4djhA	0.12	0.20	0.87	1.17	Download
10	7dceX	0.11	0.20	0.89	1.28	Download

Tables

Table S1. The compound information for different growth months (Q5-Q9) detected in UHPLC-MS/MS analysis.

Alignment ID	Metabolite name	Rt(min)	Expreiment Mz	Peak area					Superclass	Class
				Q5	Q6	Q7	Q8	Q9		
NEG27104	Ginsenoside Rf	10.76	845.49	161556532.53	55854856.64	121120046.40	82651209.76	43818260.31	Lipids and lipid-like molecules	Glycerolipids
NEG3881	Sedoheptulose	1.23	209.07	1102983826.75	2117588807.07	1846355849.79	2264709843.41	10447155545.23	Organic oxygen compounds	Organooxygen compounds
NEG8237	Homoeriodictyol	7.68	301.13	1597419.56	8693278.29	24912033.39	6573359.70	118714552.94	Phenylpropanoids and polyketides	Flavonoids
NEG16263	Phlorhizin	8.66	481.14	29674770.91	42319437.69	42180146.67	8680641.97	4457333.75	Phenylpropanoids and polyketides	Flavonoids
POS13263	Hematoxylin	7.77	303.09	47103858.87	130932317.27	82862181.19	15161171.41	9353255.66	Organoheterocy	Benzopyrans

									clic compounds	
NEG18059	Centaurein	8.71	521.13	24740246.40	23683869.29	262935741.68	76950223.95	317442273.09	Phenylpropanoids and polyketides	Flavonoids
POS25687	Iridin	8.79	523.14	24046477.45	5605074.06	18227689.43	9983942.35	4923160.24	Phenylpropanoids and polyketides	Isoflavonoids
POS2442	Cinnamaldehyde	11.11	133.06	19009942.04	583020304.12	148169917.53	619320130.39	1882063713.37	Phenylpropanoids and polyketides	Cinnamaldehydes
NEG7858	FA 18:3	12.58	293.21	274947013.25	442291297.26	150415786.96	138607643.16	95815829.18	Lipids and lipid-like molecules	Fatty acids
POS3305	cis-Anethole	7.34	149.10	218525920.71	263332752.92	368459350.27	357255647.90	314711733.26	Benzenoids	Phenol ethers
POS2441	Asparagine	1.38	133.06	175881242.43	422200037.07	32222407.99	91879877.16	15112724.54	Organic acids and derivatives	Carboxylic acids and derivatives
POS1612	Proline	1.47	116.07	25998152452.32	3703131223.22	5044797109.99	5748951919.66	2966908499.78	Organic acids and derivatives	Carboxylic acids and

										derivatives
NEG2635	Myoinositol	1.21	179.06	2261577407.00	806540343.11	11630009565.81	12981222793.53	10821511411.01	Organic oxygen compounds	Organooxygen compounds
POS4851	Citrulline	1.36	176.10	345505585.46	114966186.50	65328807.20	108842852.98	58156118.13	Organic acids and derivatives	Carboxylic acids and derivatives
POS24210	caudatin	11.22	491.30	166038033.94	47820171.99	45686708.14	45982420.31	38253188.69	Lipids and lipid-like molecules	Steroids and steroid derivatives
POS21665	Ophiopogonoside A	8.69	441.25	381247014.35	23010720.47	29972429.11	29134821.88	57875628.00	Lipids and lipid-like molecules	Prenol lipids
POS19762	Artesunate	8.85	407.17	333413132.43	374580682.42	103627234.73	169814288.41	164176023.19	Lipids and lipid-like molecules	Prenol lipids
POS16205	Syringetin	10.07	347.08	74391564.44	8672490.46	29556092.18	14299616.08	11147505.55	Phenylpropanoids and polyketides	Flavonoids
POS20444	Icariside F2	7.06	420.19	536271993.02	369840424.77	504641898.81	1019332496.18	701401673.29	Organic oxygen	Organooxygen

									compounds	compounds
POS22841	Forsythoside E	7.27	463.18	36961978.13	33077040.81	140233901.10	135575923.59	94316944.54	Organic oxygen compounds	Organooxygen compounds
POS25523	Ganoderic Acid B	10.02	519.33	38758241.52	111440333.58	213506406.68	160186593.91	160861358.41	Lipids and lipid-like molecules	Prenol lipids
POS15000	Dihydromelilotoside	7.12	329.12	46002203.28	83042009.41	246234737.69	229820553.91	191331209.76	Organic oxygen compounds	Organooxygen compounds
POS21972	Gaultherin	7.80	447.15	1242720.01	1233743.57	22546531.61	1615475.17	64511492.04	Organic oxygen compounds	Organooxygen compounds
NEG15327	Apiopaeonoside	9.02	459.15	26007878.08	64414437.82	45362065.81	99688305.34	625449289.24	Organic oxygen compounds	Organooxygen compounds
POS31776	[(2R,3R,4S,5R,6S)-3,4,5-tris(acetyloxy)-6-[(5,8-dihydroxy-1,4-dioxo-3-{[(2S,3R,4S,5R,6R)-3,4,5-tris(	6.61	883.21	26981927.43	34373183.33	44219385.24	5267662.82	1742994.38	Benzenoids	Naphthalenes

	acetyloxy)-6-[(acetyloxy)methyl]oxan-2-yl]oxy}-1,4-dihydronaphthalen-2-yl)oxy]oxan-2-yl]methyl acetate									
NEG3246	Gluconic acid	0.93	195.05	95319294996.70	10965372485.94	68532463556.59	18550431089.49	9575670443.62	Organic oxygen compounds	Organooxygen compounds
POS17847	Geniposidic acid	7.37	373.11	9692321.76	10651905.47	3432963.54	2747765.85	4886529.01	Lipids and lipid-like molecules	Prenol lipids
POS20216	Deoxycholic acid	11.13	415.28	1153176834.02	1312876036.18	1366551641.51	851425531.52	803342925.45	Lipids and lipid-like molecules	Steroids and steroid derivatives
POS11295	Indole-3-acetyl-valine	7.81	275.14	111256666.62	19731770.57	14373511.38	11153556.86	2493028.02	Organic acids and derivatives	Carboxylic acids and derivatives
POS21859	Lamiide	7.60	445.13	136866465.19	72671284.06	33694957.06	103157031.79	13481354.81	Lipids and lipid-like molecules	Prenol lipids

POS2639	p-Anisaldehyde	8.33	137.06	225861447.38	143235220.66	184768627.31	210392631.82	640778267.32	Benzenoids	Benzene and substituted derivatives
NEG18812	Olivil 4'-O-Glucoside	7.89	537.20	4058161.21	3941553.69	3755850.62	12060056.82	314639339.19	Lignans, neolignans and related compounds	Lignan glycosides
POS22401	Asperulosidic acid	1.46	455.12	3244961551.2 3	1899491532.7 1	3305537533.0 5	1905207398.6 6	701044564.68	Lipids and lipid-like molecules	Prenol lipids
POS18886	Loganin	6.58	391.16	20179665.21	15933150.34	95419740.52	133144175.83	96436651.52	Lipids and lipid-like molecules	Prenol lipids
POS24195	6'-O-beta-D-Apio furanosylswerosi de	7.13	491.18	103342145.76	175011565.07	590113120.80	545786490.66	402250881.18	Organic oxygen compounds	Organooxyg en compounds
NEG11736	Hexahydrocurcu min	9.78	373.17	81731776.51	62357172.93	181158013.89	145975121.69	766082945.76	Phenylpropanoid s and polyketides	Diarylheptan oids
POS14945	N-Fructosyl phenylalanine	6.24	328.14	3682710805.3 6	1113448152.56	272277283.69	986489880.79	6926010.06	Organic acids and derivatives	Carboxylic acids and

										derivatives
POS3981	4-Methylcoumarin	7.15	161.06	961922144.77	1083587113.87	388979725.86	785508515.58	407502497.19	Phenylpropanoids and polyketides	Coumarins and derivatives
POS5678	Trimethyllysine	1.23	189.16	29952122.97	3845230.06	11774080.75	7172305.04	8595761.57	Organic acids and derivatives	Carboxylic acids and derivatives
POS17994	3-(3,4-dihydro-2H-benzo[b][1,4]dioxepin-7-yl)-7-ethoxy-2-methyl-4H-chromen-4-one	6.98	375.12	288548827.14	415130828.86	507505430.93	128170634.25	51825588.62	Phenylpropanoids and polyketides	Isoflavonoids
NEG10767	6:3+6O fatty acyl hexoside	1.03	353.07	529761663.37	289356968.22	383491394.30	359903201.14	32358418.46	Lipids and lipid-like molecules	Fatty acids
NEG9592	Vanilloyl glucose	6.27	329.09	252586539.49	489454566.69	190854258.76	93777794.20	142239750.49	Phenylpropanoids and polyketides	Tannins
NEG2994	Pyroglutamic acid	3.00	188.06	229592652.82	71598863.60	222761856.87	108000604.67	28351580.11	Organic acids and derivatives	Carboxylic acids and derivatives
NEG2369	Acetylleucine	7.66	172.10	83687329.94	53794638.72	154630648.99	106708451.00	218745932.51	Organic acids	Carboxylic

									and derivatives	acids and derivatives
POS18665	1-O-Sinapoylglucose	7.37	387.13	13621586.09	17189951.06	121613456.84	46375271.72	156947914.25	Phenylpropanoids and polyketides	Cinnamic acids and derivatives
NEG11645	Syringin	8.57	371.13	201859375.43	61641170.55	39371530.83	23956632.25	20305972.65	Organic oxygen compounds	Organooxygen compounds
POS15843	Myricanol	9.43	341.17	139484134.11	60301557.01	145825872.68	113288186.64	363095269.14	Phenylpropanoids and polyketides	Diarylheptanoids
NEG6950	Dihydroresveratrol	12.21	275.09	107919.80	18965094.53	9027214.10	23647666.90	80522557.37	Phenylpropanoids and polyketides	Stilbenes
POS25877	(Z)-9-(3-hydroxy-4-methoxyphenyl)-2-(2,4,5-trimethoxybenzylidene)-8,9-dihydro-2H-furo[2,3-f]chromene-3,7-dione	7.50	527.13	2139860.82	1388.42	1388.42	1388.42	1388.42	Phenylpropanoids and polyketides	Diarylheptanoids
POS21046	Grayanotoxin I	8.60	430.28	117768199.17	16310047.41	33689640.15	18179086.72	4339356.13	Lipids and	Prenol lipids

									lipid-like molecules	
POS25646	Taurochenodeoxycholic acid	9.55	522.29	26311000.49	19041667.76	47981465.51	98936023.36	80855535.09	Lipids and lipid-like molecules	Steroids and steroid derivatives
POS24285	Carminic acid	8.30	493.10	7563542.88	11040344.60	14764625.43	6068564.24	175096442.37	Benzenoids	Anthracenes
NEG12790	Scopolin	7.65	399.09	94638012.47	327517077.11	174915980.45	584643002.80	596836894.00	Phenylpropanoids and polyketides	Coumarins and derivatives
POS23279	Asiatic acid	9.54	471.35	357799584.02	664559894.53	1227029230.27	1137139275.06	1087054265.91	Lipids and lipid-like molecules	Prenol lipids
POS28263	Benzoylpaeoniflorin	6.81	607.18	17944735.62	742939.49	6268628.23	35476.27	87092.86	Lipids and lipid-like molecules	Prenol lipids
POS4982	Methionine ethyl ester	6.31	178.09	91449363.55	39941785.64	72500476.35	20402404.67	11216122.20	Phenylpropanoids and polyketides	Isoflavonoids
POS3940	2-(4-aminotetrahydro-2H-pyran-4-yl)acetic acid	7.65	160.10	32892309.94	11000577.46	8126606.65	12181754.76	7954718.08	Organic oxygen compounds	Organooxygen

										compounds
NEG22177	Linarin	7.28	637.18	10415892.26	8860890.69	12870094.70	9488397.16	47942287.94	Phenylpropanoids and polyketides	Flavonoids
POS16467	Bergenin	1.46	351.07	1297304233.84	1997717973.50	2186997182.86	3205482045.22	2132823274.40	Benzenoids	Benzene and substituted derivatives
NEG6159	7,8-Dihydroyangonin	8.79	259.12	363561730.39	823922377.30	533115241.08	149205829.89	140967549.08	Kavalactones	Kavalactones
POS4901	Hymecromone	11.11	177.05	179440458.95	5212805529.71	1293790252.37	5766627567.59	17258152608.35	Phenylpropanoids and polyketides	Coumarins and derivatives
POS15356	Leonurine	7.25	334.14	76015612.11	31319202.30	35854137.64	40663611.95	35496919.68	Benzenoids	Benzene and substituted derivatives
POS13199	beta-D-Glucopyranosiduronic acid, 4-methylphenyl	6.69	302.12	66477812.85	123015311.40	63055057.68	165960279.22	176059013.58	Organic oxygen compounds	Organooxygen compounds
NEG20586	Baccatin III	9.17	585.23	237764201.93	307649408.71	32625614.50	53578582.53	83846108.48	Lipids and lipid-like	Prenol lipids

									molecules	
NEG14806	Quercitrin	8.40	447.09	8494272156.47	2522945780.71	2469848678.06	2326392346.31	2839677040.76	Phenylpropanoids and polyketides	Flavonoids
POS17947	2-((4-ethyl-8,8-dimethyl-2-oxo-2,8,9,10-tetrahydropyrano[2,3-f]chromen-5-yl)oxy)-N-isopropylacetamide	11.85	374.20	26568600.05	1671110.01	7146539.98	11630352.16	137580334.62	Phenylpropanoids and polyketides	Coumarins and derivatives
POS20343	4'-Demethylepipodophyllotoxin	6.65	418.15	288483964.77	18954212.13	122108771.21	1703915.26	5133907.74	Lignans, neolignans and related compounds	Lignan lactones
POS14041	Dehydrophytosphingosine - 2H	9.48	314.27	254358443.99	154008524.03	227039877.01	122150394.44	164594006.58	Organic nitrogen compounds	Organonitrogen compounds

Notes: The tables are arranged in descending order of OPLSDA.VIP size.The core metabolites with VIP ≥ 1 were selected from VirtualTaste and were used to predict important explanatory variables for sweet and bitter substances.Q5-Q9 refers to the samples of the leaves of C. paliurus collected from May to September,2023

Table S2. Molecular docking results of ligands with T1R2\T1R3 and T2R4\T2R14

Metabolite name	OPLSDA.VIP	BitterDB	sweet	Prediction	bitter	Prediction
			Category 1			
Ganoderic Acid B	1.119371849	Yes	0.787	active	0.806	active
Deoxycholic acid	1.192314781	Yes	0.743	active	0.968	active
Indole-3-acetyl-valine	1.349165461	Yes	0.741	active	0.726	active
			Category 2			
Ginsenoside Rf	1.138605755	Yes	0.998	active	0.994	inactive
FA 18:3;O	1.268509538	Yes	0.915	active	0.872	inactive
cis-Anethole	1.145374868	Yes	0.911	active	0.838	inactive
Icariside F2	1.04386449	Yes	0.796	active	0.771	inactive
Forsythoside E	1.122004667	Yes	0.791	active	0.744	inactive
Gluconic acid	1.10560069	Yes	0.749	active	0.88	inactive
Olivil 4'-O-Glucoside	1.202634393	Yes	0.715	active	0.778	inactive
			Category 3			
Centaurein	1.227749339	No	0.924	active	0.526	active
Iridin	1.049658686	No	0.924	active	0.526	active
Syringetin	1.178014358	No	0.804	active	0.581	active
Dihydromelilotoside	1.315207118	No	0.784	active	0.598	active
Lamiide	1.088654791	No	0.733	active	0.597	active
			Category 4			
Sedoheptulose	1.215268387	No	0.982	active	0.965	inactive
Hematoxylin	1.010290164	No	0.934	active	0.781	inactive
Asparagine	1.010406649	No	0.895	active	0.92	inactive
Proline	1.206744481	No	0.874	active	0.803	inactive
Citrulline	1.280906204	No	0.841	active	0.721	inactive
Gaultherin	1.135968087	No	0.766	active	0.744	inactive
Apiopaeonoside	1.283996895	No	0.755	active	0.773	inactive
